# Primary systemic vasculitis in children in Estonia: a retrospective study from 2001-2010

**DOI:** 10.1186/1546-0096-9-S1-P93

**Published:** 2011-09-14

**Authors:** Sirje Tarraste, Mari Laan, Jaanika Illison, Chris Pruunsild

**Affiliations:** 1 Tallinn Children`s Hospital, Tartu University Department of Pediatrics, Estonia

## Background

Most primary systemic vasculitides (PSV) in children are relatively rare diseases, but can have a significant morbidity and mortality. The two most common PSV are Henoch-Schönlein purpura (HSP) and Kawasaki disease (KD).

## Aim of the study

To analyze the occurence, clinical presentation and outcome of PSV diagnosed in Estonia last 10 years. In cases of HSP and KD the presenting features and the main treatment options were described.

## Methods

Retrospective review of medical charts of PSV patients hospitalized to Tallinn and Tartu Children’s Hospitals from 2001 to 2010. All PSV patients (except HSP) are diagnosed in these two tertiary children’s hospitals in Estonia.

## Results

197 new cases of PSV were diagnosed during these years – HSP in 154, KD in 35, Churg-Staruss syndrome in 2, and polyarteritis nodosa in 3 cases, respectively. Wegener granulomatosis, microscopic polyangiitis and hypocomplementaemic urticarial vasculitis were all diagnosed in one patient. The age distribution ranged from 3 months to 16 years, in cases of KD – from 2 months to 13 years. 38% of all patients with HSP received short term corticosteroids. 91,4 % patients with KD received high dose intravenous immunoglobulin. Three children died during the follow - up period (Wegener granulomatosis, polyarteritis nodosa, microscopic polyangiitis).

## Conclusions

We determined the number of new cases of PSV within a hospital based population in Estonia. Despite the small population of Estonia we had the overall variety of PSV. HSP was the most common vasculitis, followed by KD. Other PSV were very rare in children in Estonia.

